# Heat stress changes mineral nutrient concentrations in *Chenopodium quinoa* seed

**DOI:** 10.1002/pld3.384

**Published:** 2022-02-06

**Authors:** Jose C. Tovar, Jeffrey C. Berry, Carlos Quillatupa, S. Elizabeth Castillo, Lucia Acosta‐Gamboa, Noah Fahlgren, Malia A. Gehan

**Affiliations:** ^1^ Donald Danforth Plant Science Center St. Louis MO USA

**Keywords:** elemental profile, heat, ionomics, nutrient composition, quinoa, seed

## Abstract

Quinoa is a popular seed crop, often consumed for its high nutritional quality. We studied how heat stress in the roots or the shoots of quinoa plants affected the concentrations of 20 elements (aluminum, arsenic, boron, calcium, cadmium, cobalt, copper, iron, potassium, magnesium, manganese, molybdenum, sodium, nickel, phosphorous, rubidium, sulfur, selenium, strontium, and zinc) in quinoa seed. Elemental concentrations in quinoa seed were significantly changed after an 11‐day heat treatment during anthesis. The type of panicle (main, secondary, and tertiary) sampled and the type of heat treatment (root only, shoot only, or whole plants) significantly affected elemental profiles in quinoa seed. Plants were also divided into five sections from top to bottom to assess the effect of panicle position on seed elemental profiles. Plant section had an effect on the concentrations of arsenic, iron, and sodium under control conditions and on copper with heat treatment. Overall, the time of panicle development in relation to the time of heat exposure had the largest effect on seed elemental concentrations. Interestingly, the quinoa plants were exposed to heat only during anthesis of the main panicle, but the elemental concentrations of seeds produced after heat treatment ended were still significantly changed, indicating that heat stress has long‐lasting effects on quinoa plants. These findings demonstrate how the nutritional quality of quinoa seeds can be changed significantly even by relatively short heat spells.

## 1 INTRODUCTION

Knowing the elemental composition of plant‐based foods is important to accurately estimate daily intake in people's diets and to avoid nutrient deficiencies or toxicities. Recommended daily consumption for minerals varies on the basis of the target population (Bost et al., 2016; Welch, 2002; World Health Organization, 2005). Differences in cultivation practices and environments can significantly affect nutrient content in crops (Assefa et al., 2019; Balboa et al., 2018; Johansson et al., 2020; Welch, 2002), potentially affecting fertilization and planting time recommendations for crops (Assefa et al., 2019; Balboa et al., 2018; Bindraban et al., 2020; Havlin, 2020; Johansson et al., 2020).

Quinoa is a grain crop prized for its high nutritional quality (Choukr‐Allah et al., 2016; Repo‐Carrasco et al., 2003; Vega‐Gálvez et al., 2010) and is reported to have high contents of many mineral nutrients, including calcium, magnesium, sodium, phosphorous, iron, copper, and zinc (Repo‐Carrasco et al., 2003). Quinoa also grows in poor soils with low water availability and high salinity (Aguilar & Jacobsen, 2003; Al‐Naggar et al., 2017; Hinojosa, González, et al., 2018; Jacobsen et al., 2003). There is increasing interest in expanding quinoa cultivation (Bazile et al., 2016; Choukr‐Allah et al., 2016; Hinojosa, González, et al., 2018; Jacobsen, 2003; Maliro et al., 2017; Pulvento et al., 2010), which will inevitably include areas with higher daily average temperatures than its native range of cultivation and excessive heat events are common. However, temperatures over 32°C cause significant physiological and phenotypic changes in quinoa resulting in lower seed yield (Bazile et al., 2016; Bertero & Ruiz, 2008; Hinojosa, Matanguihan, & Murphy, 2018; Lesjak & Calderini, 2017; Tovar et al., 2020), with the most pronounced effects happening when plants are exposed to heat during anthesis (Lesjak & Calderini, 2017).

Heat stress has been demonstrated to have a variety of effects on plant nutritional profiles, depending on the species and tissues examined. (Sehgal et al., 2018; Soares et al., 2019). In quinoa, temperature affects the nutrient composition of both straw (Matías, Cruz, & Reguera, 2021) and seed (Matías, Rodríguez, et al., 2021). Quinoa seeds have higher protein and fiber content and lower fat (Matías, Rodríguez, et al., 2021) and carbohydrate (Matías, Rodríguez, et al., 2021; Vrancheva et al., 2019) content with cultivation at higher temperatures. The concentration of nitrogen, phosphorous, and potassium in seeds was higher in a hotter year (20°C to 35°C during flowering) than in a cooler year (12°C to 30°C during flowering) of cultivation, whereas calcium content was lower in the hotter year, and magnesium content was not significantly different (Matías, Rodríguez, et al., 2021).

We previously showed that heat treatment during anthesis of the main panicle has a significant impact on plant development and seed yield (Tovar et al., 2020). We expand on these findings to demonstrate how heat stress during anthesis of the main panicle can affect nutrient composition in seeds at plant maturity. We report the effects of controlled heat stress on the concentrations of 20 elements: boron (B), sodium (Na), magnesium (Mg), aluminum (Al), phosphorous (P), sulfur (S), potassium (K), calcium (Ca), manganese (Mn), iron (Fe), cobalt (Co), nickel (Ni), copper (Cu), zinc (Zn), arsenic (As), selenium (Se), rubidium (Rb), strontium (Sr), molybdenum (Mo), and cadmium (Cd) in the seeds of the genome‐sequenced quinoa accession QQ74 (Jarvis et al., 2017). We show how differential heat stress in the roots versus the shoot of the plant, panicle type, and panicle position within the plant impact seed elemental concentrations.

## 2 RESULTS AND DISCUSSION

**FIGURE 1:**
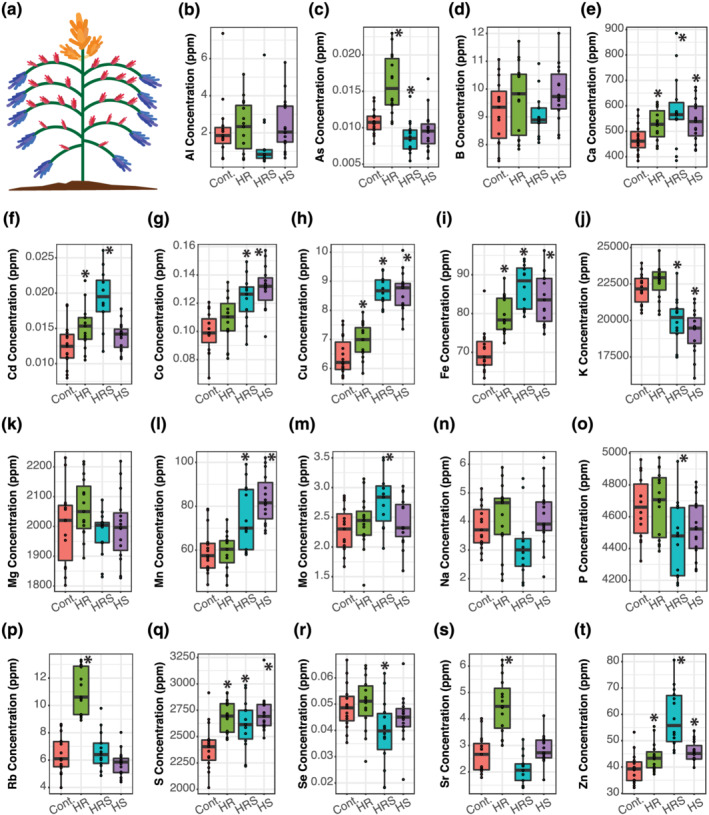
Main panicle seed elemental concentrations. (a) Distribution of the three panicle types along a quinoa plant: Main panicle is shown in orange, secondary panicles are shown in blue, and tertiary panicles are shown in red. (b–t) Concentration of 19 elements. An asterisk over a box indicates significant difference from roots and shoots held at 22°C (Cont.) (Wilcoxon rank‐sum test *p* value <.05). HR, heated roots, with roots held at 30°C and shoots held at 22°C; HRS, heated roots and shoots, with roots at held at 30°C and shoots held at 35°C; HS, heated shoots, with shoots held at 35°C and roots held at 22°C. For sample sizes, please see Table S1

The elemental composition of quinoa seed was analyzed for 20 elements: B, Na, Mg, Al, P, S, K, Ca, Mn, Fe, Co, Ni, Cu, Zn, As, Se, Rb, Sr, Mo, and Cd in main, secondary, and tertiary panicles (Figure 1a). The analyzed seeds were harvested from plants grown under constant control conditions or plants that returned to control conditions after an 11‐day heat treatment started at first anthesis (Tovar et al., 2020). The four temperature treatments were as follows: (1) control, with plants growing at 22°C; (2) heated roots (HR), with roots growing at 30°C and shoots growing at 22°C; (3) heated shoots (HS), with roots growing at 22°C and shoots growing at 35°C; and (4) heated roots and shoots (HRS), with roots growing at 30°C and shoots growing at 35°C. To test if the elemental accumulation in seed was impacted by panicle type, we harvested seeds independently from each main and secondary panicle, whereas all tertiary panicles were pooled per plant to reach sampling quantities with repetitions required for elemental profiling. For further information on the methodology used for heat treatments and elemental composition analysis, please refer to Section 4. Raw data resulting from ionomics analysis can be found in Data S1.

### 2.1 Elemental accumulation in seed is affected by the time of panicle development relative to the time of heat exposure

In quinoa, main panicles emerge and develop first, then secondary panicles, and lastly tertiary panicles. We have observed that quinoa accession QQ74 main panicles emerge during an early stage of the plant's life cycle, approximately 50 to 60 days after sowing (DAS), with the majority of main panicle development happening before 100 DAS. Secondary panicles develop approximately between 60 and 130 DAS. Tertiary panicles develop approximately between 80 and 150 DAS, overlapping more closely with the development of secondary panicles than with main panicles. Plants were heat‐treated for 11 days. The first day of heat treatment was between 60 and 65 DAS (depending on the moment of first anthesis), and the last day of heat treatment was 11 days later. Therefore, main and secondary panicles were exposed to heat at different developmental stages, and our heat treatment was not applied to tertiary panicles.

**FIGURE 2:**
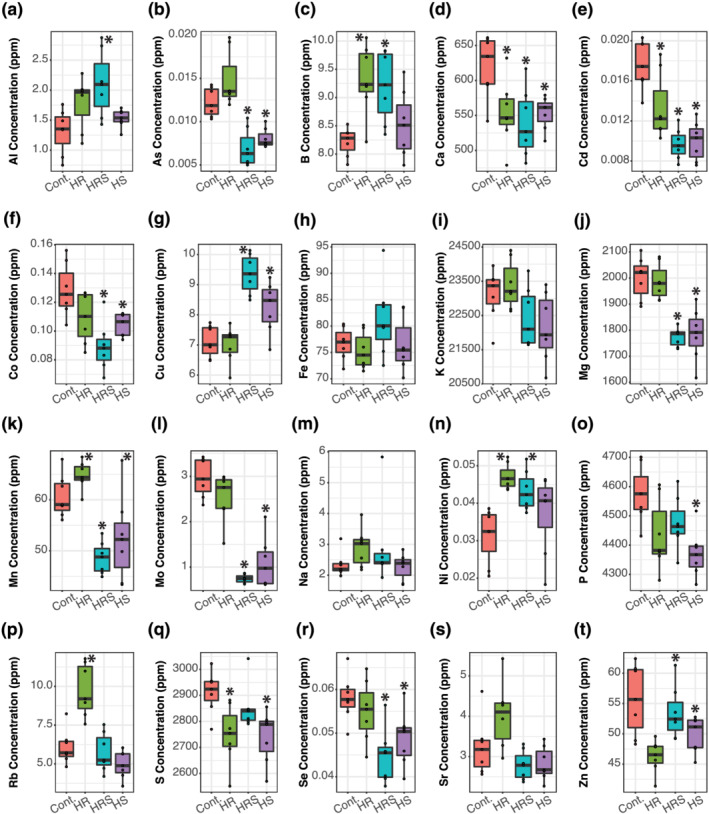
Secondary panicle seed elemental concentrations. (a–t) Concentration of 20 elements. An asterisk over a box indicates significant difference from roots and shoots held at 22°C (Cont.) (Wilcoxon rank‐sum test *p* value <.05). HR, heated roots, with roots held at 30°C and shoots held at 22°C; HRS, heated roots and shoots, with roots at held at 30°C and shoots held at 35°C; HS, heated shoots, with shoots held at 35°C and roots held at 22°C. For sample sizes, please see Table S1

**FIGURE 3:**
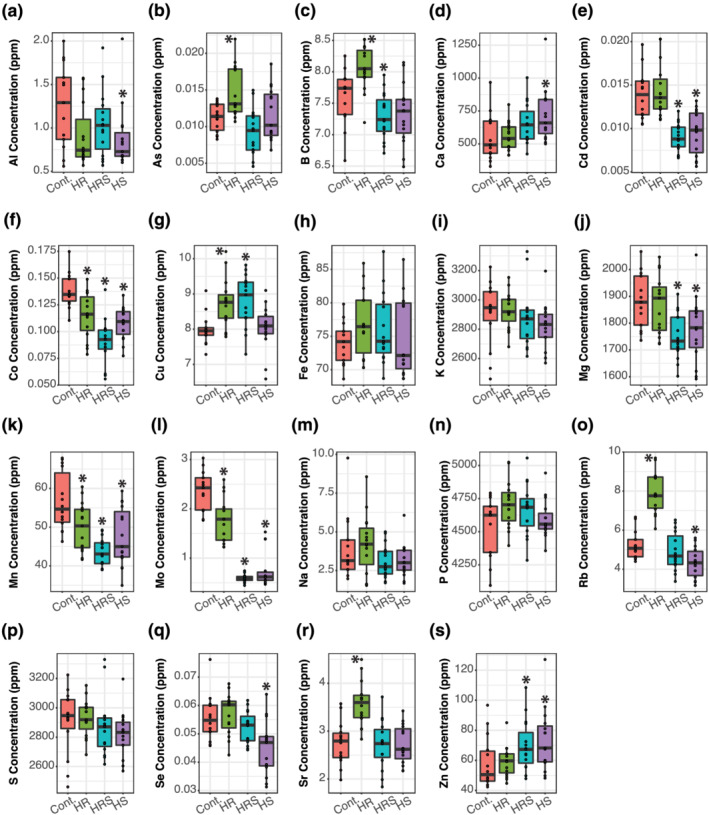
Tertiary panicle seed elemental concentrations. (a–s) Concentration of 19 elements. An asterisk over a box indicates significant difference from roots and shoots held at 22°C (Cont.) (Wilcoxon rank‐sum test *p* value <.05). HR, heated roots, with roots held at 30°C and shoots held at 22°C; HRS, heated roots and shoots, with roots at held at 30°C and shoots held at 35°C; HS, heated shoots, with shoots held at 35°C and roots held at 22°C. For sample sizes, please see Table S1

Analysis of the concentrations of the 20 elements in main, secondary, and tertiary panicle seeds showed that the type of panicle had a significant impact on elemental accumulation. In main panicle seeds (Figure 1), heat treatments significantly changed the concentrations of 15 elements (As, Ca, Cd, Co, Cu, Fe, K, Mn, Mo, P, Rb, S, Se, Sr, and Zn) when compared with control seeds (Wilcoxon rank‐sum test *p* values <.05), whereas the concentrations of four other elements (Al, B, Mg, and Na) did not significantly change after heat treatment (Wilcoxon rank‐sum test *p* values >.05). In secondary panicles' seeds (Figure 2), heat treatments significantly changed the concentrations of 16 elements (Al, As, B, Ca, Cd, Co, Cu, Mg, Mn, Mo, Ni, P, Rb, S, Se, and Zn) when compared with control seeds (Wilcoxon rank‐sum test *p* values <.05), whereas the concentrations of four other elements (Fe, K, Na, and Sr) did not significantly change (Wilcoxon rank‐sum test *p* values >.05). In tertiary panicles' seeds (Figure 3), heat treatments significantly changed the concentrations of 15 elements (Al, As, B, Ca, Cd, Co, Cu, Mg, Mn, Mo, Ni, Rb, Se, Sr, and Zn) when compared with control seeds (Wilcoxon rank‐sum test *p* values <.05), whereas the concentrations of five other elements (Fe, K, Na, P, and S) did not significantly change (Wilcoxon rank‐sum test *p* values >.05). Although Ni concentration was measured in all seed samples, the concentrations were too small to accurately quantify in both main and tertiary panicles, so Ni was dropped from the main panicle and tertiary panicle seed analyses. For a complete list of *p* values resulting from comparing each element's concentration between each heat treatment (HR, HRS, and HS) and control, sample sizes, and summary statistics, please refer to Table S1.

**FIGURE 4:**
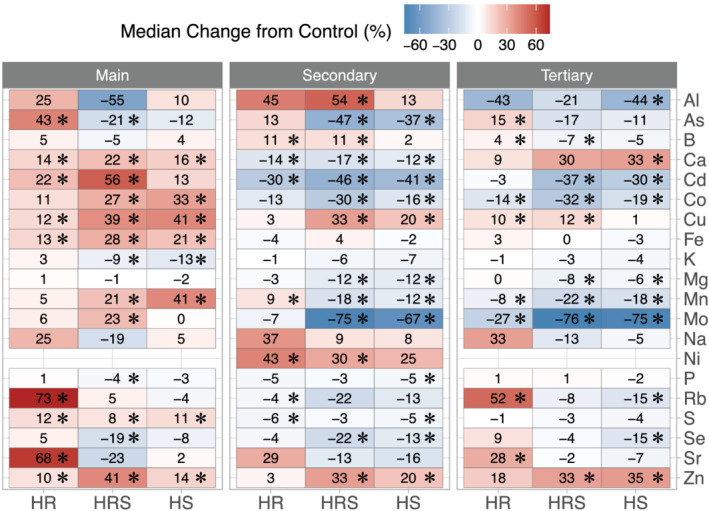
Median percent changes in seed elemental concentrations for heated roots, with roots held at 30°C and shoots held at 22°C (HR), heated roots and shoots, with roots at held at 30°C and shoots held at 35°C (HRS), and heated shoots, with shoots held at 35°C and roots held at 22°C (HS), relative to roots and shoots held at 22°C (control), for main, secondary, and tertiary panicles. An asterisk inside a box indicates significant difference from control (Wilcoxon rank‐sum test *p* value <.05). For sample sizes, please see Table S1

A side‐by‐side comparison of how heat treatments changed elemental concentrations in the three panicle types shows that secondary and tertiary panicles responded more similarly in seed elemental concentration changes than main panicles (Figure 4). In main panicles, heat treatments significantly increased the concentrations of 44% of elements on average, whereas in secondary and tertiary panicles, only 17% and 16% of elements concentrations were significantly higher after heat treatment, respectively. The difference between main and secondary and tertiary panicle seeds was also evident in elements that had lower concentrations after heat treatment. In main panicles, only 9% of elements had significantly lower concentrations after heat treatment, whereas in secondary and tertiary panicles, 37% and 28% had significantly lower concentrations after heat treatment, respectively. Therefore, although heat treatment in the main panicle resulted in mostly higher elemental concentrations than in control, in secondary and tertiary panicles, elemental concentrations tended to be lower in heat‐treated plants than in control.

The effect of panicle type we measured can be ascribed to two different factors: (1) position of the panicle along the plant and (2) time of panicle development relative to period of heat exposure. Huber et al. (2016) had previously shown significant effects from position along the plant on elemental profiles of soybean seeds and leaves. The similar responses in elemental profiles to heat treatment between secondary and tertiary panicles (Figure 4) may be due to their overlapping developmental times, to their spatial proximity in the plant (Figure 1a), or both.

Interestingly, because heat treatment was applied during anthesis of the main panicle, seed development and elemental accumulation in nearly all seeds occurred after heat treatment ended. We previously reported that there are lasting effects of heat treatment on development and yield (Tovar et al., 2020). Similarly, heat treatment has significant effects in seed elemental composition that last through the plant's life, even when the plant is no longer exposed to heat.

### 2.2 Heating roots and heating shoots result in different seed elemental profiles

Similar to panicle type, the type of heat treatment (HR, HRS, or HS) significantly affected seed elemental concentrations. HR caused significantly higher concentrations in 30% of elements and significantly lower concentrations in 12% of elements than control across all panicle types. HRS caused 28% significantly higher and 29% significantly lower elemental concentrations than control across all panicle types. HS resulted in 19% elements with significantly higher concentrations and 32% elements with significantly lower concentrations than control across all panicles (Figure 4). Overall, HR induced higher elemental concentrations more frequently, whereas HS more frequently induced lower elemental concentrations than control, and HRS induced higher and lower elemental concentrations at nearly the same rate. For *p* values resulting from comparing each element's concentration between each heat treatment (HR, HRS, and HS) and control, summary statistics, and sample sizes, please see Table S1.

The different effects observed in seed elemental concentrations in response to root versus shoot heating could indicate that elemental uptake from the soil (defined as elements moving from the soil to the epidermis of the roots), or transport within the plant, may be affected by heat. The elements that were affected by root heating but not by shoot heating (significant differences from control seen in HR only or in both HR and HRS, Figure 4) were all at low concentrations (<10 ppm): As, Cd, Rb, and Sr in main panicles; Al, B, Ni, and Rb in secondary panicles; and As, B, Cu, and Sr in tertiary panicles. It is interesting that although we previously reported that HR treatment did not have a significant impact on plant development or seed yield (Tovar et al., 2020), HR had a significant effect on seed elemental composition. However, because no element was only affected by root heating but not by shoot heating in all three panicle types, the effects of heat on elemental uptake from the soil to the root epidermis could not be differentiated from the effects of heat on element transport within the plant.

The concentration of As is significantly increased by HR treatment in main and tertiary panicles. The median As concentration in main panicles was 0.015 and 0.013 μg g^−1^ in tertiary panicles, with the highest As concentration measured at 0.024 μg g^−1^. This is well below the maximum allowed As concentration in cereals of 0.1 μg g^−1^ established by the FDA (U.S. Food and Drug Administration, 2020).

Elements that were affected only by shoot heating but not by root heating (significant differences from control seen in HS only or in both HRS and HS, Figure 4) were Co, Cu, Fe, K, and Mn in main panicles; As, Co, Cu, Mg, Mo, P, Se, and Zn in secondary panicles; and Ca, Cd, Mg, Se, and Zn in tertiary panicles. Similarly to elements that responded only to root heating, the elements that responded only to shoot heating were not the same across all three panicle types. Lastly, the elements that showed significant changes in concentration from both root and shoot heating combined (HRS only) but did not show significant effects from either root (HR) or shoot heating (HS) alone were Mo and P in main panicles and Al in secondary panicles. No elements showed an effect only from heating the entire plant (HRS) in tertiary panicle seed. Because no single element showed an effect only in HS or in HRS across all three panicle types, the effects of heat on element transport within the plant could not be differentiated from the effects of panicle type.

Although the type of heat treatment had an effect on seed elemental composition, no elements were consistently affected by any one of the three heat treatments tested across all three panicle types. Although various studies have shown evidence that root temperature affects the concentrations of different elements in other plant species (Ambebe et al., 2010; Schwartz et al., 1987; Tindall et al., 1990; Turner & Lahav, 1985; Yan et al., 2012), it remains unclear if heat treatment significantly affects element uptake from the soil to the root epidermis in quinoa. Altogether, our results suggest that the larger effect in seed elemental concentration is likely determined by elemental transport within the plant, as well as timing of panicle development relative to the timing of the heat stress.

### 2.3 The location of the panicles in the plant plays a significant role in determining the concentrations of 8 out of 20 elements after heat exposure

**FIGURE 5:**
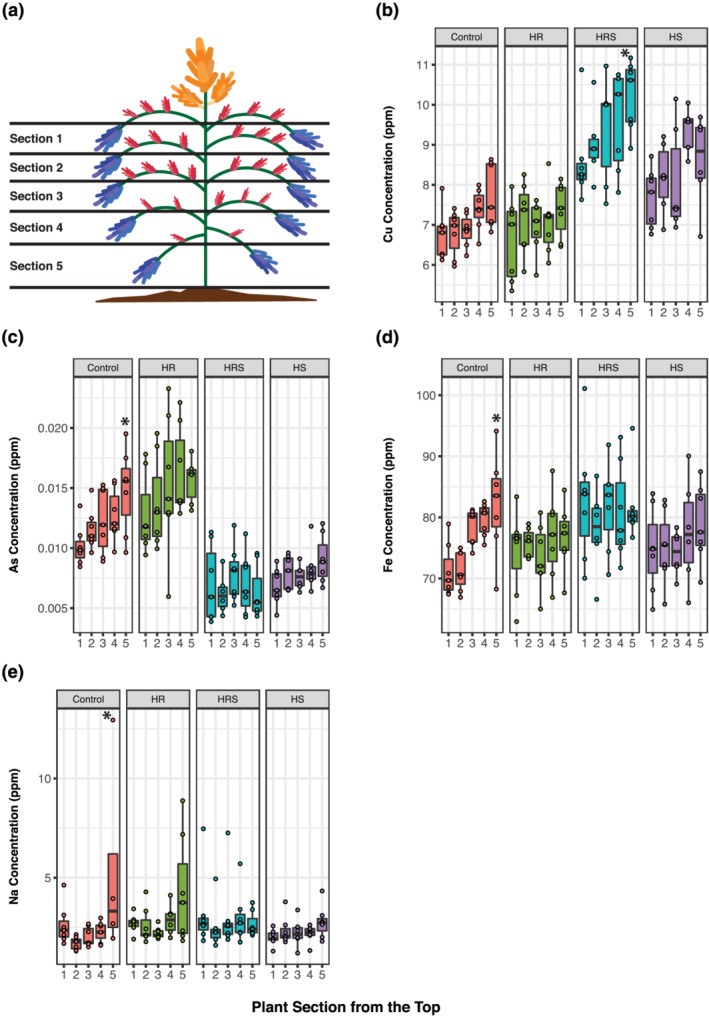
Secondary panicle seed elemental concentrations for each plant section. (a) Quinoa plant showing how the five plant sections were divided for this analysis. (b) Cu concentration. (c) As concentration. (d) Fe concentration. (e) Na concentration. An asterisk over a box indicates a significant difference from Section 1 of the same treatment (*t* test with Tukey adjustment *p* value <.05). Control, roots and shoots held at 22°C; HR, heated roots, with roots held at 30°C and shoots held at 22°C; HRS, heated roots and shoots, with roots at held at 30°C and shoots held at 35°C; HS, heated shoots, with shoots held at 35°C and roots held at 22°C. For sample sizes, please see Table S2. For exact contrast *p* values, please see Table S3

Analysis of elemental composition both by panicle type and by the type of treatment indicates a potential effect of panicle position along the plant on seed elemental profiles. To assess the effect of panicle position in the plant on seed elemental profiles, we divided each plant into five sections, numbered from top to bottom (Figure 5a). Because main panicles are located only at the top of the plant and tertiary panicles' seed was harvested all together per plant, this analysis was done only in seed harvested from secondary panicles. Secondary panicle seed was assigned to one of five plant sections (Data S1, column panicle_position) based on the respective branch number (Data S1, column panicle_id), counting branches from top to bottom of the plant.

For the three heat treatments (HR, HRS, and HS) and 20 elemental concentrations measured in seed, panicle position only showed a significant effect on Cu concentration in Section 5 (Figure 5), the section at the bottom of the plant, under the HRS treatment. The median Cu concentration increased by 28% from Section 1 to Section 5. A second analysis was done to detect influences of plant sections on elemental concentrations by testing for trends along plant sections, as a significant nonzero slope (Table S4). A trend along plant sections was significant in 21.7% of heat treatments (*t* test for nonzero slope *p* value <.05). The elements with significant trends along plant sections in heat treatments were As in HR and HS, Co in HR and HS, Cu in HRS and HS, Fe in HS, Mo in HR and HS, Na in HRS, S in HR and HS, and Zn in HS. Under control treatment, panicle position had a significant effect only in Section 5 for elements As, Fe, and Na (Figure 5). The median changes in these three elements from Sections 1 to 5 were as follows: As increased by 59%, Fe increased by 11%, and Na increased by 41%. A significant trend along plant sections was found for control treatment in As, Cu, Fe, Na, and Se (25% of elements). All other elemental concentrations did not significantly change in seed (Figure S1). For a complete list of sample sizes and summary statistics per section, element, and treatment, please see Table S2. For the *p* values resulting from contrasting the different sections within treatments and elements, please see Table S3. For the *p* values resulting from testing for nonzero slopes along sections within treatments and elements, please see Table S4.

Our results show that the effect of panicle position along the plant on seed elemental profiles can be significant but only in 8 out of 20 elements analyzed. Huber et al. (2016) previously showed that soybean canopy and pod position had a significant effect on the concentrations of Mg, Fe, and Cu. Similar to what we observed in seed, Mg, Fe, and Cu concentrations in soybean seeds and leaves at the bottom of the plant were higher than at the top of the plant (Huber et al., 2016). This supports our finding that panicle position on the plant can affect element concentration in almost half (8 out of 20) of the elements.

Under heat, our results show that the position of the panicle along the plant is not a significant factor in seed elemental profiles, with the sole exception of Cu under HRS treatment. Cu can be toxic at high concentrations (>20 to 100 μg g^−1^) both for the plant and for human consumption (Gupta & Gupta, 1998; Påhlsson, 1989; Zheng et al., 2007). Although Cu concentration increased significantly under HRS treatment, the highest median concentration we measured was under 11 μg g^−1^ in Section 5 (Figure 5 and Table S2). This is well below toxicity levels and lower than a high median reported in maize (Bost et al., 2016). Although higher Cu intake (below toxicity levels) is generally good for nutrition (Bost et al., 2016; Soares et al., 2019), this shift in seed Cu concentration could be a concern for quinoa cultivation in soils with exceptionally high Cu availability, where toxicity levels might be reached.

Quinoa is frequently studied for its high tolerance to salt (Iqbal et al., 2020; Koyro et al., 2008; Ruiz et al., 2016; Schmöckel et al., 2017) and often proposed as an interesting crop for cultivation in regions of the world with high salinity soils (Adolf et al., 2013; Bazile et al., 2016; Choukr‐Allah et al., 2016; Grenfell‐Shaw & Tester, 2021; Hinojosa, González, et al., 2018; Iqbal et al., 2020). Quinoa seeds store approximately sixfold more Na when grown on saline soils (González et al., 2021), indicating that seeds may serve as a Na sink under saline conditions. The lower part of a quinoa plant is where leaves senesce first, and these senescing leaves have been shown to act as Na sinks (Iqbal et al., 2020). Our data show that the seeds in the lower panicles also store more Na under control conditions, suggesting that quinoa seeds are also acting as Na sinks in lower parts of the plant (Section 5). However, our results also show that seeds do not accumulate as much Na in the lower part of the plant after heat exposure, suggesting a potential interaction between heat and salinity tolerance. This is interesting because soils with high salinity often occur in regions where high temperature spells are common (Abuelgasim & Ammad, 2019; Becker et al., 2017; Choukr‐Allah et al., 2016; HanumanthaRao et al., 2016). Therefore, it would be interesting to expand existing controlled combined heat and salt stress studies in quinoa (Becker et al., 2017) to measure effects on yield and nutrient composition in different genotypes, as well as more broadly study the effects of the combined heat and salt stresses on the respective known tolerance mechanisms.

### 2.4 Panicle type and shoot heating cause the largest changes in seed elemental concentrations

**FIGURE 6:**
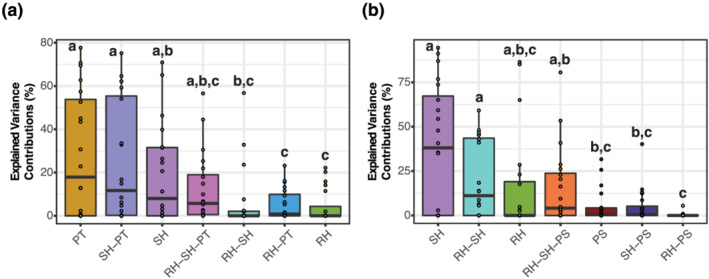
Contributions to the model variance in seed elemental concentrations explained by the factors analyzed in this work. (a) Contributions to the model variance in seed elemental concentrations by the factors panicle type (PT), interaction between shoot heating and panicle type (SH–PT), shoot heating (SH), the interaction between root heating, shoot heating and panicle type (RH–SH–PT), interaction between root heating and shoot heating (RH–SH), interaction between root heating and panicle type (RH–PT), and root heating (RH). (b) Contributions to the model variance in seed elemental concentrations by the factors shoot heating (SH), interaction between root heating and shoot heating (RH–SH), root heating (RH), interaction between root heating, shoot heating and plant section (RH–SH–PS), plant section (PS), interaction between shoot heating and plant section (SH–PS), and interaction between root heating and plant section (RH–PS). Letters above boxes represent statistical significance at *p* < .05 from a *t* test. For exact *t*‐test *p* values resulting from pairwise comparisons between these factors, please see Table S5. All sample sizes were *n* = 20

To estimate the contributions to seed elemental concentrations of the factors we tested (panicle type, root and shoot heating, and plant section), we measured the effects of each factor on the variance of seed elemental concentrations. We found that for Ni, Cd, Fe, S, P, Al, Na, Zn, Se, and Ca, most of the variance (>50%) was unexplained by our model, meaning that the majority of variance was not due to panicle type, root heating, shoot heating, or plant section (Figure S2). However, partial correlations of Rb, Mo, Cu, Mn, Sr, K, As, Mg, B, and Co indicate that panicle type, root heating, shoot heating, and plant section explained most of the model variance in seed concentrations. To identify which of the factors we measured accounted for most of the explained variance across all elements measured, we estimated partial correlations for these four factors and averaged them per factor. This analysis supported our conclusion that panicle type had a larger impact than heat treatment, with the interaction between panicle type and shoot heating causing a similar change in seed elemental concentrations (Figure 6a). Plant section was analyzed separately from panicle type, in secondary panicles only, because of sampling requirements and also because panicle type and plant section are not independent factors. This analysis confirmed that the plant section and root heating were the two factors that induced the smallest changes in seed elemental concentrations, with shoot heating producing larger changes than panicle type and root heating (Figure 6b). Overall, panicle type had the largest effect on seed elemental concentrations, followed by shoot heating, and lastly by root heating and plant section. Because panicle type is the combination of both the position of the panicle along the plant and the time of panicle development relative to period of heat exposure, these variance measurements support our conclusion that the time of panicle development relative to period of heat exposure is the major contributing factor to changes in seed elemental concentration.

## 3 CONCLUSIONS

This study shows that exposure of quinoa plants to heat affects the contents of many elements in seed produced from the same plants. We conclude that the main factor affecting seed elemental profiles after heat exposure is the timing of panicle development in relation to the period of heat exposure, whereas the panicle position on the plant, and the plant part (roots or shoots) exposed to heat had smaller effects, on fewer elements.

Interestingly, our heat treatment only lasted 11 days during anthesis of the main panicle, but we observed significant elemental composition changes in seed produced after heat treatment ended. Although we previously showed that a relatively brief heat treatment can have lasting effects on seed yield (Tovar et al., 2020), we have now shown that these same relatively short heat treatments can significantly affect seed elemental profiles. Therefore, this suggests that even short heat spells can affect the nutritional quality of quinoa seed, a change that could impact different stakeholders, including quinoa growers and consumers.

## 4 EXPERIMENTAL PROCEDURES

### 4.1 Seed materials and heat treatments

Seeds were collected from plants from four different heat treatments, as described in Tovar et al. (2020). Briefly, plants of quinoa accession QQ74 (PI 614886) were grown until first anthesis at 22°C, 50% relative humidity, 400 μmol m^−2^ s^−1^, in a 12‐h day photoperiod. Then 15 plants were placed into each of four different temperature treatments: (1) control, with plants growing at 22°C; (2) heated roots, with roots growing at 30°C and shoots growing at 22°C; (3) heated shoots, with roots growing at 22°C and shoots growing at 35°C; and (4) heated roots and shoots, with roots growing at 30°C and shoots growing at 35°C. Temperatures were kept constant during both day and night. The soil temperature was 30°C when the air temperature was 35°C inside growth chambers. Therefore, 30°C was used as the heat treatment for roots. We previously showed that the same heat treatments used in this study result in significantly lower yield and delayed maturity than control when shoots were heated, but not when roots were heated (Tovar et al., 2020); however, we were interested in seeing if this root heat treatment could affect mineral nutrient profiles. Plants were treated for 11 days, and then all plants were placed in a greenhouse set to 22°C until harvest. Seeds were then collected from each panicle for main and secondary panicles, and all tertiary panicles were collected per plant. Seed was cleaned using an air blast seed cleaner (ABSC; ALMACO, Nevada, IA) followed by manual cleaning with a mesh sifter. Seed was stored in paper bags at room temperature until ionomics analysis.

### 4.2 Ionomics analysis

The concentrations (in mg of element per kg of seed) of B, Na, Mg, Al, P, S, K, Ca, Mn, Fe, Co, Ni, Cu, Zn, As, Se, Rb, Sr, Mo, and Cd in seed were measured through ionomics analysis. Approximately 50 mg of seeds was weighed for each sample (Data S1). Three samples were weighed from each panicle. Ionomic analysis was performed using inductively coupled plasma mass spectrometry by the ionomics facility at the Donald Danforth Plant Science Center, as described in Pauli et al. (2018). Briefly, each seed sample was digested overnight at room temperature in 2.5‐ml nitric acid with 20‐ppb indium as a sample preparation standard. The next day, samples were heated to 100°C for 3 h and then diluted to 10 ml with ultrapure water. Samples were then diluted 5× with ultrapure water containing 2‐ppb yttrium as an instrument standard, using an inline ESI PrepFAST auto dilution system. Element concentrations were measured in a PerkinElmer ELAN 6000 DRC‐e mass spectrometer. Element concentrations obtained from the instrument were then corrected for run‐to‐run variation with the yttrium and indium standards, and a matrix matched control, which was run every 10 samples.

### 4.3 Data statement

The data used for this study are in Data S1. Data were analyzed in RStudio Version 1.4.1103 running R Version 4.5. The R script used to analyze the data is available at https://github.com/danforthcenter/quinoa-ionomics-2021.

### 4.4 Outlier detection and removal

Identification of influential observations using Cook's distance (Cook, 1977) was done using two approaches. First, principal component analysis was performed using all elemental concentrations (Data S1), and whole samples were removed from all analyses if the sample influence was greater than three times the mean influence when regressed for treatment on Principal Component 1. Second, the same procedure was done on individual element concentrations that were not removed in the first approach, to account for effects from the ionomics analysis.

### 4.5 Inference of treatment effects

The Wilcoxon rank‐sum test (Mann & Whitney, 1947) was used to establish statistically significant differences between treatments (*p* value <.05) in seed elemental concentrations, with the base R function pairwise.wilcox.test using no *p*‐value correction (p.adjust.method = “none”) because the number of total comparisons is less than 20.

### 4.6 Inference of plant section effects

To determine if there are differences across the sections of a plant, samples from individual secondary panicles were grouped into five sections of the plant. Sections were numbered 1 to 5 from top to bottom of the plant (Figure 5a). A generalized linear mixed‐effect model was created with treatment and treatment–section interaction terms as fixed effects, and a random intercept to account for individual plant effects. Significantly nonzero slopes along plant sections (*p* value <.05) were determined with a *t* test of the interaction between treatment and plant sections. Mean separation using lsmeans, from the lsmeans R package (Lenth, 2016), was used to compare sections within treatments. Significantly nonzero differences (*p* value <.05) were identified through *t*‐test comparisons with Tukey adjustment of the plant sections within treatments.

### 4.7 Estimation of contributions to variance in seed elemental concentrations of panicle type, heat treatment, and plant section

The effects of (1) panicle type (PT), (2) root heating (RH, whether the roots were heated or not), and (3) shoot heating (SH, whether the shoots were heated or not) on the variance in elemental concentrations in seed were estimated using a variance component model considering all interactions between the three factors (RH–PT, RH–SH, SH–PT, and RH–SH–PT) on the concentrations of all 20 elements, with the lmer package in R. These effects were estimated on the entire data set, after outlier removal. The effects of (1) plant section (PS), (2) RH, and (3) SH and their interactions (RH–PS, RH–SH, SH–PS, and RH–SH–PS) were analyzed in the same manner but exclusively on the data from secondary panicles, because secondary panicles were the only panicle type divided into plant sections. Therefore, panicle type was not included in the model that had plant section as a factor. Using these models, the variance contribution of each factor on seed elemental concentrations was estimated as a percent of the total variance. The variance not explained by any of the factors in the models was considered unexplained. The explained, or model, variance was defined as the total variance minus the unexplained variance. Partial correlations of each factor are calculated as a percentage of the explained variance indicating their respective contributions. Finally, a *t* test was used to establish statistically differences between the contributions of each factor to the explained variance.

#### SIGNIFICANCE STATEMENT

This study shows that heat stress in quinoa during anthesis of the main panicle results in altered seed elemental profiles at harvest. Seed elemental profiles were mostly influenced by the timing of panicle development relative to the time of heat exposure. The type of heat treatment (root vs. shoot heating) and the position of the panicle along the plant had a smaller but statistically significant effect on seed elemental profiles.

## CONFLICT OF INTEREST

The authors declare that they have no conflicts of interest.

## AUTHOR CONTRIBUTIONS

J. C. T. and M. A. G. conceived the research; J. C. T. designed the experiments; J. C. T., J. C. B., and L. A.‐G. analyzed the data; J. C. T., C. Q., and S. E. C. performed the experiments; J. C. T., J. C. B., and M. A. G. wrote the article; and J. C. T., J. C. B., M. A. G., L. A.‐G., and N. F. edited the article.

## Supporting information


**Figure S1.** Elemental concentrations in secondary panicle seed by plant section (1 to 5 counting from the top of the plant, Figure 5a) for: (a) Al, (b) B, (c) Ca, (d) Cd, (e) Co, (f) K, (g) Mg, (h) Mn, (i) Mo, (j) Ni, (k) P, (l), Rb, (m) S, (n) Se, (o) Sr, and (p) Zn. Control, roots and shoots held at 22 °C; HR, heated roots, with roots held at 30 °C and shoots held at 22 °C; HS, heated shoots, with shoots held at 35 °C and roots held at 22 °C; HRS, heated roots and shoots, with roots held at 30 °C and shoots held at 35 °C. For sample sizes please see Table S1.

**Figure S2.** Contributions to variance in seed elemental concentrations. (a) Contributions to variance in seed elemental concentrations by the factors root heating (RH), panicle type (PT), shoot heating (SH), interaction between root heating and panicle type (RH‐PT), interaction between root heating and shoot heating (RH‐SH), interaction between shoot heating and panicle type (SH‐PT), interaction between root heating, shoot heating and panicle type (RH‐SH‐PT), as well as the variance not explained by any of these factors (Unexplained). (b) Contributions to variance in seed elemental concentrations by the factors root heating (RH), plant section (PS), shoot heating (SH), interaction between root heating and plant section (RH‐PS), interaction between root heating and shoot heating (RH‐SH), interaction between shoot heating and plant section (SH‐PS), interaction between root heating, shoot heating and plant section (RH‐SH‐PS), as well as the variance not explained by any of these factors (Unexplained) in secondary panicles.

**Data S1.** Raw data resulting from ionomics analysis. Element name abbreviations: B11: boron, Na23: sodium, Mg26: magnesium, Al27: aluminum, P31: phosphorous, S34: sulfur, K39: potassium, Ca44: calcium, Fe54: iron, Mn55: manganese, Co59: cobalt, Ni60: nickel, Cu63: copper, Zn66: zinc, As75: arsenic, Se78: selenium, Rb85: rubidium, Sr88: strontium, Mo98: molybdenum, Cd111: cadmium.

**Table S1.** Sample sizes, summary statistics and p‐values for the comparisons between each heat treatment (HR, HRS, and HS) and control for every panicle type, treatment and element analyzed.

**Table S2.** Sample sizes and summary statistics for every plant section, treatment and element analyzed.

**Table S3.** Plant section contrasts' p‐values for every treatment and element analyzed.

**Table S4.** T‐test statistics and p‐values for testing non‐zero slopes along plant sections for every treatment and element analyzed.

**Table S5.** T‐test statistics and p‐values for every comparison between factors shown in Figure 6.
