# Supplementary material for: Heat stress changes mineral nutrient concentrations in Chenopodium quinoa seed
Source: Plant Direct. 2022 Feb 6;6(2):e384. doi: 10.1002/pld3.384 (PMC8818816; doi:10.1002/pld3.384)
Supplement: Supplementary file 1 — Figure S1. Elemental concentrations in secondary panicle seed by plant section (1 to 5 counting from the top of the plant, Figure 5a) for: (a) Al, (b) B, (c) Ca, (d) Cd, (e) Co, (f) K, (g) Mg, (h) Mn, (i) Mo, (j) Ni, (k) P, (l), Rb, (m) S, (n) Se, (o) Sr, and (p) Zn. Control, roots and shoots held at 22 °C; HR, heated roots, with roots held at 30 °C and shoots held at 22 °C; HS, heated shoots, with shoots held at 35 °C and roots held at 22 °C; HRS, heated roots and shoots, with roots held at 30 °C and shoots held at 35 °C. For sample sizes please see Table S1. [file PLD3-6-e384-s003.pdf]

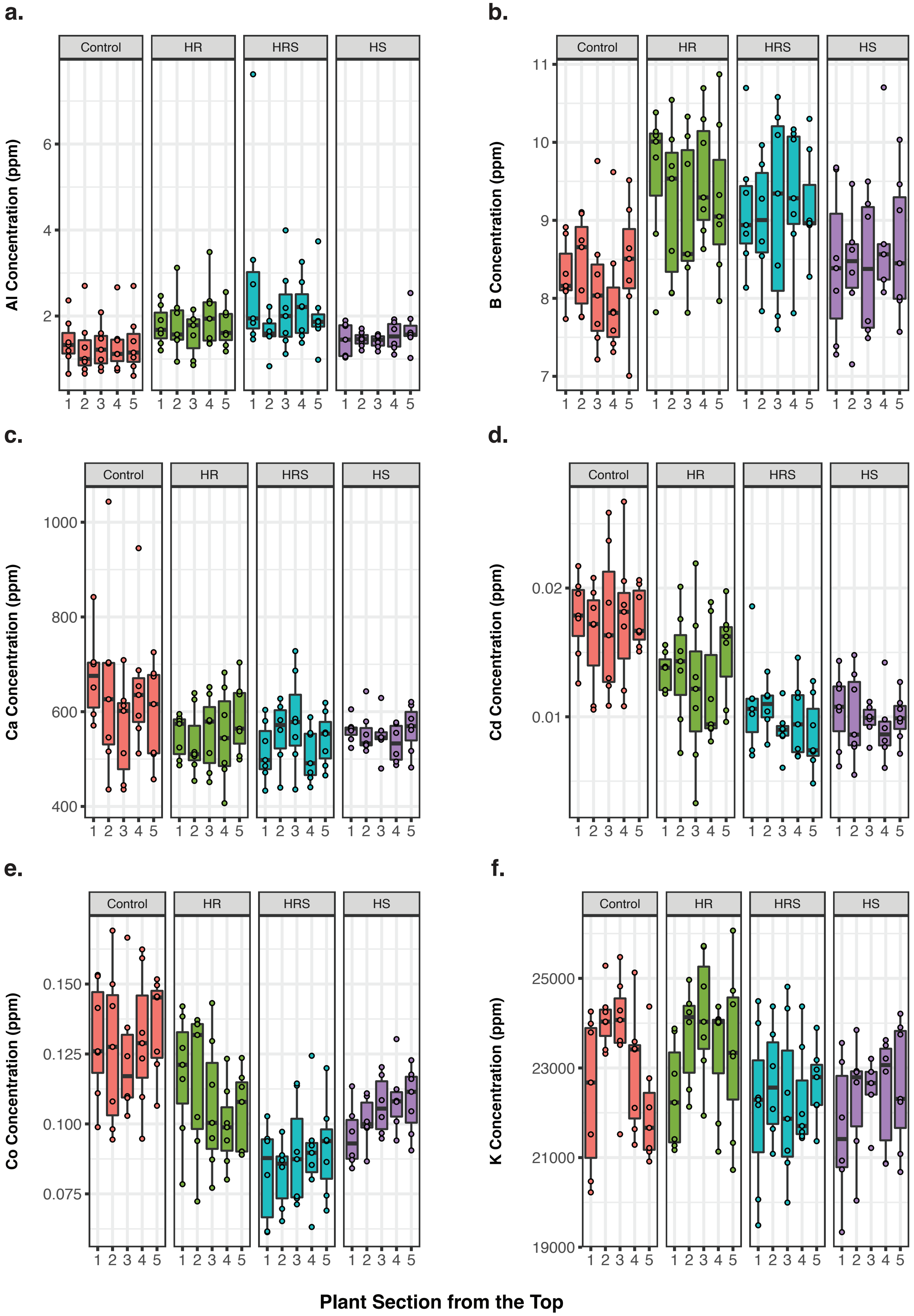
a.
Control
HR
HRS
HS
Al Concentration (ppm)
2
4
6
1 2 3 4 5
b.
Control
HR
HRS
HS
B Concentration (ppm)
7
8
9
10
11
1 2 3 4 5
c.
Control
HR
HRS
HS
Ca Concentration (ppm)
400
600
800
1000
1 2 3 4 5
d.
Control
HR
HRS
HS
Cd Concentration (ppm)
0.01
0.02
1 2 3 4 5
e.
Control
HR
HRS
HS
Co Concentration (ppm)
0.075
0.100
0.125
0.150
1 2 3 4 5
f.
Control
HR
HRS
HS
K Concentration (ppm)
19000
21000
23000
25000
1 2 3 4 5
Plant Section from the Top

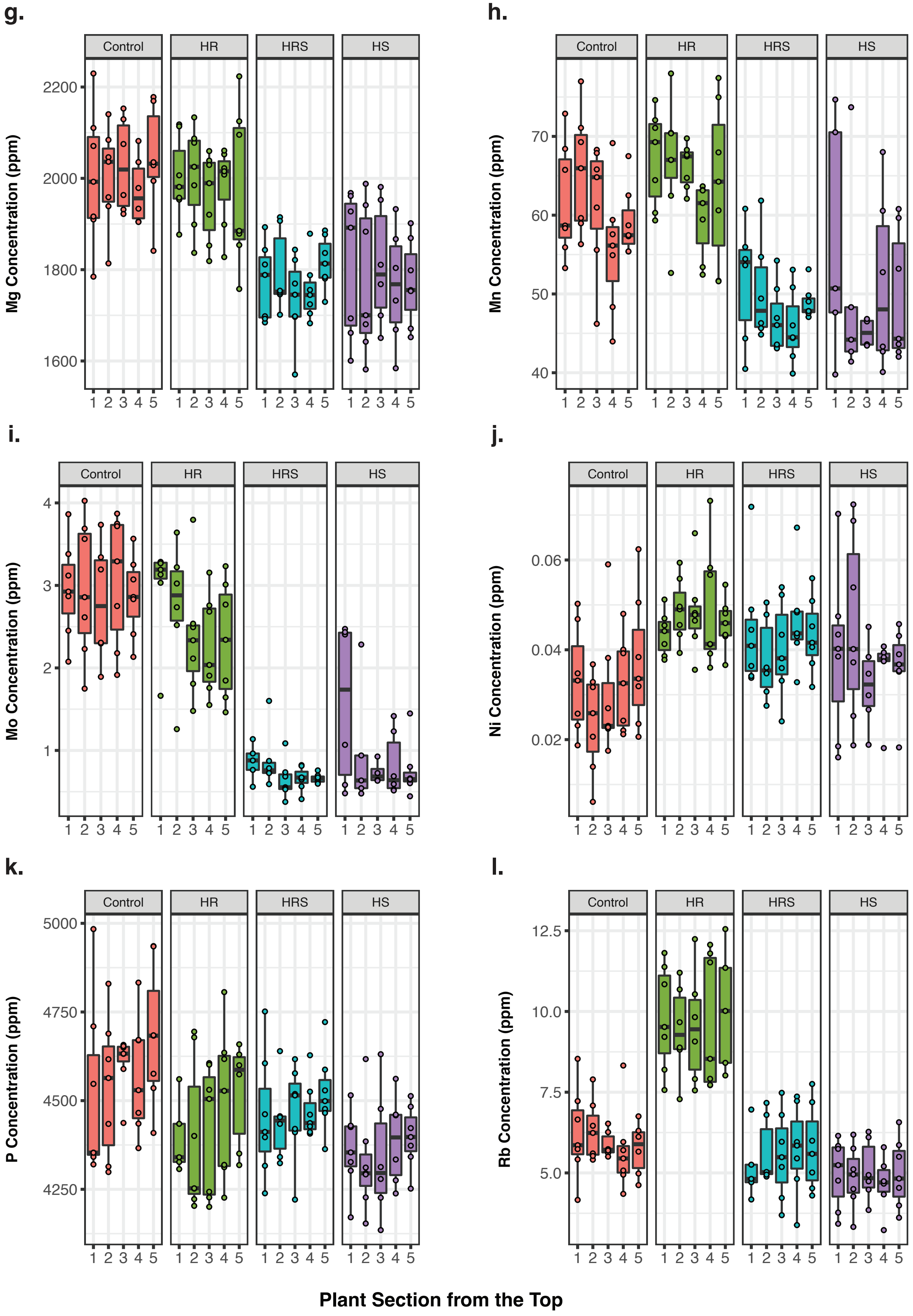
g.
Control
HR
HRS
HS
Mg Concentration (ppm)
2200
2000
1800
1600
1 2 3 4 5
h.
Mn Concentration (ppm)
70
60
50
40
i.
Mo Concentration (ppm)
4
3
2
1
j.
Ni Concentration (ppm)
0.06
0.04
0.02
k.
P Concentration (ppm)
5000
4750
4500
4250
l.
Rb Concentration (ppm)
12.5
10.0
7.5
5.0
Plant Section from the Top

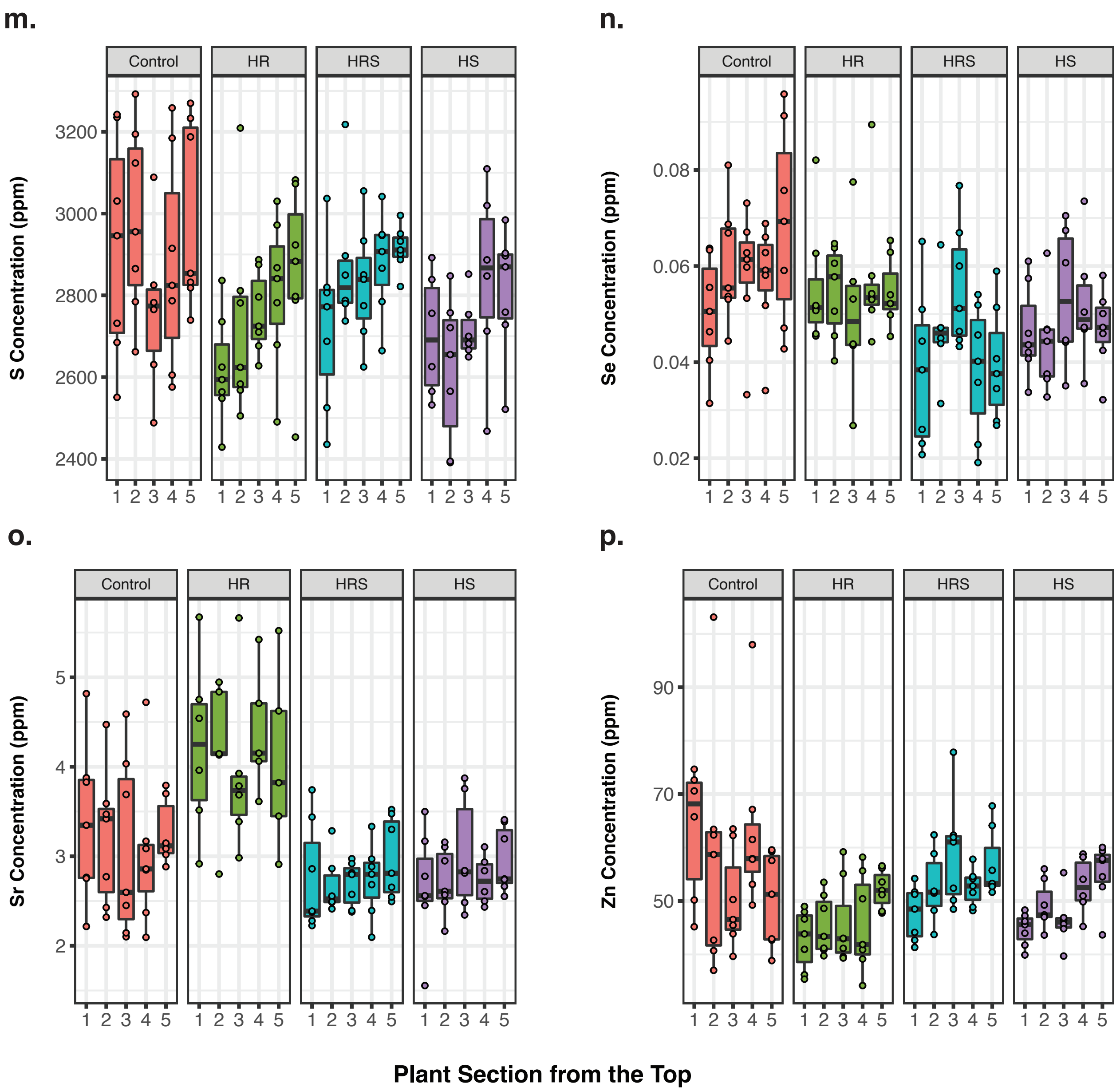

m.
Control
HR
HRS
HS
S Concentration (ppm)
3200
3000
2800
2600
2400
1 2 3 4 5
n.
Se Concentration (ppm)
0.08
0.06
0.04
0.02
o.
Sr Concentration (ppm)
5
4
3
2
p.
Zn Concentration (ppm)
90
70
50
Plant Section from the Top
