# Supplementary figures and images for: Heat stress changes mineral nutrient concentrations in Chenopodium quinoa seed
Source: Plant Direct. 2022 Feb 6;6(2):e384. doi: 10.1002/pld3.384 (PMC8818816; doi:10.1002/pld3.384)

**a.**

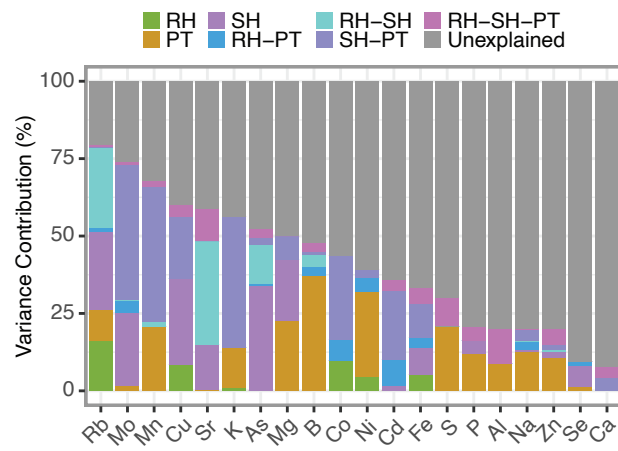

**b.**

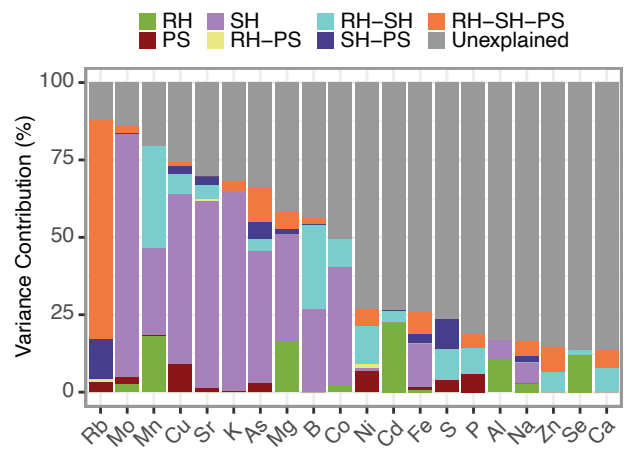

Supplement: Supplementary file 2 — Figure S2. Contributions to variance in seed elemental concentrations. (a) Contributions to variance in seed elemental concentrations by the factors root heating (RH), panicle type (PT), shoot heating (SH), interaction between root heating and panicle type (RH‐PT), interaction between root heating and shoot heating (RH‐SH), interaction between shoot heating and panicle type (SH‐PT), interaction between root heating, shoot heating and panicle type (RH‐SH‐PT), as well as the variance not explained by any of these factors (Unexplained). (b) Contributions to variance in seed elemental concentrations by the factors root heating (RH), plant section (PS), shoot heating (SH), interaction between root heating and plant section (RH‐PS), interaction between root heating and shoot heating (RH‐SH), interaction between shoot heating and plant section (SH‐PS), interaction between root heating, shoot heating and plant section (RH‐SH‐PS), as well as the variance not explained by any of these factors (Unexplained) in secondary panicles. [file PLD3-6-e384-s008.pdf]
